# N-linked glycosylation of flavivirus E protein contributes to viral particle formation

**DOI:** 10.1371/journal.ppat.1011681

**Published:** 2023-10-11

**Authors:** Kotaro Ishida, Hirokazu Yagi, Yukinari Kato, Eiji Morita

**Affiliations:** 1 Department of Biochemistry and Molecular Biology, Faculty of Agriculture and Life Science, Hirosaki University, Japan; 2 Division of Biomolecular Function, Bioresources Science, United Graduate School of Agricultural Sciences, Iwate University, Morioka, Japan; 3 Graduate School of Pharmaceutical Sciences, Nagoya City University, Nagoya, Japan; 4 Exploratory Research Center on Life and Living Systems (ExCELLS), National Institutes of Natural Sciences, Okazaki, Japan; 5 Department of Antibody Drug Development, Tohoku University Graduate School of Medicine, Sendai, Japan; 6 Department of Molecular Pharmacology, Tohoku University Graduate School of Medicine, Sendai, Japan; Duke-National University of Singapore, SINGAPORE

## Abstract

In the case of the Japanese encephalitis virus (JEV), the envelope protein (E), a major component of viral particles, contains a highly conserved N-linked glycosylation site (E: N154). Glycosylation of the E protein is thought to play an important role in the ability of the virus to attach to target cells during transmission; however, its role in viral particle formation and release remains poorly understood. In this study, we investigated the role of N-glycosylation of flaviviral structural proteins in viral particle formation and secretion by introducing mutations in viral structural proteins or cellular factors involved in glycoprotein transport and processing. The number of secreted subviral particles (SVPs) was significantly reduced in N154A, a glycosylation-null mutant, but increased in D67N, a mutant containing additional glycosylation sites, indicating that the amount of E glycosylation regulates the release of SVPs. SVP secretion was reduced in cells deficient in galactose, sialic acid, and N-acetylglucosamine modifications in the Golgi apparatus; however, these reductions were not significant, suggesting that glycosylation mainly plays a role in pre-Golgi transport. Fluorescent labeling of SVPs using a split green fluorescent protein (GFP) system and time-lapse imaging by retention using selective hooks (RUSH) system revealed that the glycosylation-deficient mutant was arrested before endoplasmic reticulum (ER)- Golgi transport. However, the absence of ERGIC-53 and ERGIC-L, ER-Golgi transport cargo receptors that recognize sugar chains on cargo proteins, does not impair SVP secretion. In contrast, the solubility of the N154A mutant of E or the N15A/T17A mutant of prM in cells was markedly lower than that of the wild type, and proteasome-mediated rapid degradation of these mutants was observed, indicating the significance of glycosylation of both prM and E in proper protein folding and assembly of viral particles in the ER.

## Author summary

N-linked glycosylation of the flavivirus E protein has been implicated in neurotoxicity and is primarily responsible for attachment to the cell surface upon the invasion of target cells. Using the HiBiT-tag system, we successfully developed a remarkably sensitive method for detecting secreted subviral particles (SVPs). Using this system, we highlighted the significant role of N-glycosylation during viral particle formation and release, particularly during prM and E protein folding and viral particle assembly. The N-glycosylation properties of E proteins within flaviviral families vary, with certain strains of West Nile Virus (WNV) or Yellow Fever Virus (YFV) lacking N-glycosylation sites. The amount of E protein released by these strains was lower than that of E proteins by viral strains containing N-glycosylation sites. This implies that the efficient assembly and release of E through N-glycosylation is a common feature among flaviviruses.

## Introduction

Flaviviruses, such as Japanese Encephalitis Virus (JEV), West Nile Virus (WNV), Zika Virus (ZIKV), Dengue Virus (DENV), Yellow Fever Virus (YFV), and Tick-Borne Encephalitis Virus (TBEV), are arthropod-borne and can cause various serious health issues in humans, including encephalitis, hemorrhagic fever, and birth defects, thus posing a significant global public health threat [1–3]. However, effective antiviral drugs have not been developed for this purpose.

Flaviviruses possess an enveloped structure and carry a plus-stranded RNA genome that encodes a single polyprotein bordered by 5’ and 3’ untranslated regions. Cleavage of this polyprotein generates three structural and seven nonstructural proteins [4]. The mature virion has a diameter of approximately 50 nm and consists of genomic RNA and a capsid enveloped in a lipid bilayer along with 90 dimers of M and E [5–8]. Structural studies have revealed the presence of at least 60 copies of capsid dimers in the virion, which play a crucial role in bridging the membrane and genomic RNA [5]. Virions are produced within the endoplasmic reticulum (ER) through budding and are subsequently transported to the extracellular environment via the conventional secretory pathway [9–11]. In addition to virions, infected cells produce a significant number of subviral particles (SVPs) comprising solely prM and E. These particles are also released from cells via the same secretory pathway as virions [12–16]. The E protein consists of three ectodomains (EDI, EDII, and EDIII), stem and transmembrane (TM) regions [17–19]. During viral entry, the E dimer transforms into a trimer through a conformational change induced by the endosomal acidic environment, exposing the EDII fusion loop to facilitate fusion between the lipid membrane and endosomal membrane of the viral particle [20–22]. The prM protein is thought to aid in proper folding of the E protein [23–26]. Moreover, it is known that the prM protein interacts with E protein to cover its fusion loop, thereby preventing the fusion of immature particles with the cell membrane during viral particle secretion [24,27,28]. Upon budding of the virion into the ER lumen, the resulting immature particles consisted of 60 prM-E heterotrimers [29–31]. During maturation, the prM protein is cleaved into pr and M by furin protease after passing through the Golgi apparatus and trans-Golgi network [28,31]. Furthermore, prM and E undergo other post-translational modifications, such as N-linked glycosylation (N-glycosylation) [32–34], and disulfide linkages [35] immediately after translation at the ER. Additionally, recent studies have demonstrated the ubiquitination of the E protein [36]. These modifications ultimately led to the formation of 90 M-E heterodimers at full maturity [7,8,37].

N-glycosylation is a post-translational modification that occurs in secreted and membrane-bound proteins and involves a consensus protein motif (N-X-S/T, where X is not proline) [38]. During N-glycosylation, a protein is initially glycosylated with oligosaccharides in the ER during translation. After trimming, the glycosylated protein is transferred to the Golgi apparatus where glycosyltransferases and glycosidases synthesize glycan structures in a stepwise manner [39]. Previous studies have shown that N-glycans in DENV particles obtained from mosquito cells are predominantly high mannose-type and galactosyl-terminated, and also contain sialic acid [40]. The analysis of glycans in TBEV particles obtained from both human and mite cells revealed the presence of galactosyl-terminated and sialic acid-containing glycans [41]. N-glycosylation sites have been identified in both prM and E proteins. The major N-glycosylation site of flaviviral E proteins is N154 [32,42], although there have been reports of strains of WNV, YFV, and ZIKV lacking this site [43–46]. In addition to N154, an asparagine residue (N67) was identified as an N-glycosylation site in the DENV E protein [47,48]. In contrast, N-glycosylation of prM is conserved among all flaviviruses, with N15 identified in JEV and WNV, N69 in DENV and ZIKV, and N13/N29 in YFV [32,49]. Non-glycosylated mutants of JEV, Murray Valley Encephalitis Virus (MVEV), WNV, and TBEV exhibit decreased neuroinvasiveness [33,50–53], whereas glycosylated ZIKV is more likely to infect the brain tissue and cause increased lethality [54–56], indicating that N-glycosylation is related to viral pathogenicity. Studies have shown that mutants with an additional glycosylation site on the JEV E protein enhance binding to cell surface lectin receptors, such as DC-SIGN [33], indicating that N-glycosylation of the virus particle plays a role in its entry into target cells. Mutations in the N-glycosylation sites also decrease viral particle production [34,49,50,57–60]. These findings suggest that N-glycosylation may also play a role in virus particle formation and release, but the stage of flavivirus release and type of N-glycosylation required for virus production remain unclear.

In this study, we introduced two types of short peptide tags, the HiBiT-tag [61] for highly sensitive detection and the GFP11-tag [62] for fluorescent labeling of the JEV E protein. This allowed us to track the viral particles during virion release and clarify the role of N-glycosylation in viral particle formation and release.

## Results

### The N-glycosylation of the E protein is essential for the release of sub-viral particles (SVPs)

**Fig 1:**
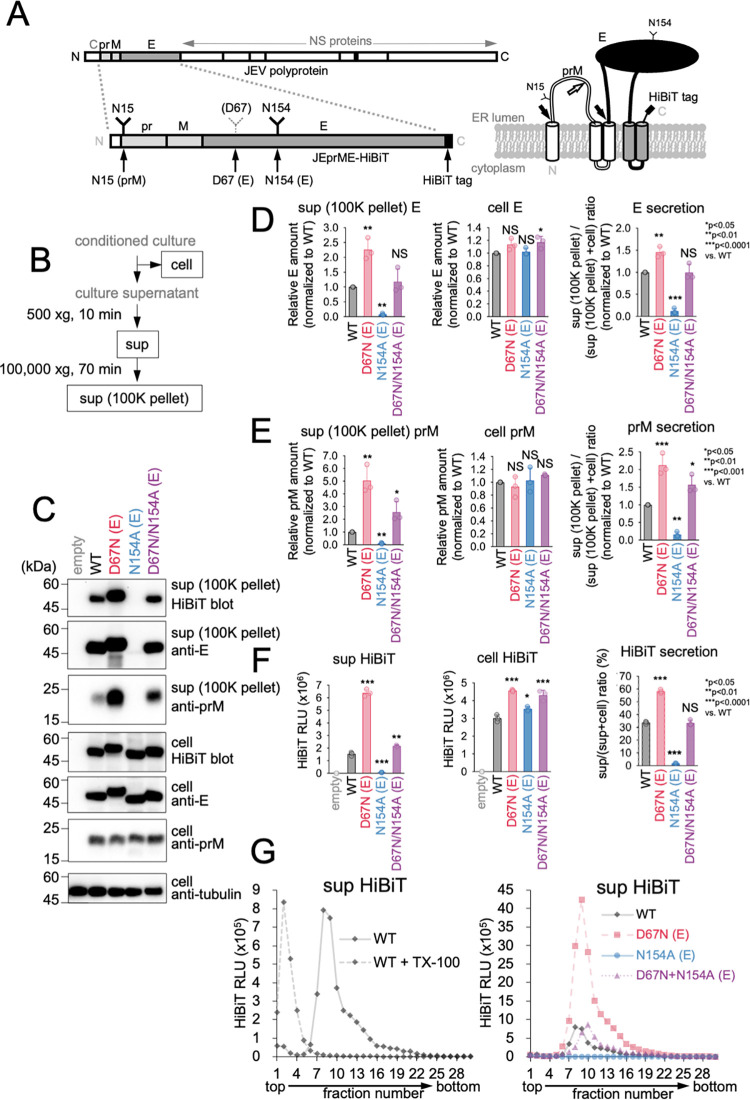
N-linked glycosylation of the E protein is essential for the release of sub-viral particles (SVPs). (A) Primary structure of the JEV polyprotein and the domain arrangement of its E protein (upper left), N-glycosylation site mutations in the JEprME-HiBiT construct (lower left), and membrane topology and cleavage site of the prME-HiBiT structural proteins on the endoplasmic reticulum membrane (lower right). The host signal peptidase cleaves at the signal peptide-prM and -prM-E junctions (black arrow), and the host furin protease cleaves at the pr-M junction (white arrow). (B) Schematic drawing of the experimental setup. (C) Immunoblotting analysis of JEV SVP release. 293T cells transfected with the indicated prME-HiBiT constructs were cultured for 48 hours, and then cells and culture supernatant were harvested. The amounts of E protein, prM protein, and alpha-tubulin in the cell lysate (cell) and in the 100,000 xg precipitate fraction of culture supernatant (100K pellet) were analyzed via immunoblotting using anti-E protein (second and fifth), anti-prM protein (third and sixth), and anti-alpha-tubulin antibodies (bottom), respectively. E protein was also detected with HiBiT blotting (top, forth). (D and E) Quantification of E (D) or prM (E) proteins in the 100K pellet (left) and cell (middle). Graphs show quantification of the immunoblots in (C). Secretion rates of E and prM proteins (right) were calculated by dividing the amount of the 100K pellet by the total E and prM amount (100K pellet + cell), respectively. (F) HiBiT-dependent NanoLuc luciferase activity (HiBiT activity) measurement analysis of JEV SVP release. HiBiT activity in both cell lysate (middle) and the 500 xg centrifugation fraction of culture supernatants (left) in (C) was measured. Secretion rates were determined by dividing the HiBiT value in culture supernatants by the total HiBiT value in the cell culture (sup + cell). Standard deviation is represented by error bars. *p < 0.05; **p < 0.01; ***p < 0.0001; NS, not significant. (G) Sucrose density gradient fractionation analysis of released SVPs. Culture supernatants of 293T cells expressing prME-HiBiT proteins were treated with (left panel, dotted line) or without (left panel, solid line, and right panel) 1% Triton X-100 and then subjected to 10–40% sucrose density gradient ultracentrifugation. HiBiT activities in each fraction were measured. Error bars represent standard deviation. Means and SDs from three independent experiments are presented.

To investigate the importance of N-glycosylation of the E protein on JEV particle release, an N-glycosylation-deficient mutant (N154A) of the E protein was created, and its release into the culture medium was examined. In addition, a JEV E protein mutant (D67N) was generated to include an extra N-glycosylation site at D67 position, which corresponds to the second N-glycosylation site in DENV (Fig 1A). To detect the released SVPs, we fused a HiBiT tag and a split NanoLuc luciferase to the C-terminus of prME and expressed it in cells (Fig 1A). This allowed us to measure the amount of released E protein by assessing HiBiT-dependent NanoLuc luciferase activity (HiBiT activity) in 500 xg supernatant fraction (SVP positive fraction) of the culture supernatants (Fig 1B). In cells expressing either the wild-type (WT) or the N-glycosylated mutant of the E protein, all E proteins were detected by immunoblotting analysis and HiBiT activity. However, the N154A mutant was not detected in the culture supernatant (Fig 1C–1F). These findings indicate that N-glycosylation is essential for the release of E protein. It is noteworthy that D67N exhibited a roughly 4-fold increase in secretion compared to the WT. Moreover, the additional D67N mutation, in conjunction with the N154A mutation, rescued the release impairment observed with the N154A mutation (Fig 1C–1F). These results suggest that a certain quantity of N-glycosylation is crucial for the release of the E protein. The culture supernatant was subjected to sucrose density gradient centrifugation analysis, which revealed a sedimentation peak in HiBiT activity. However, upon pretreatment of the sample with detergent, this peak disappeared, indicating that it corresponded to assembled SVPs (Fig 1G, left). The presence of a single sedimentation peak indicated a correlation between the HiBiT activity observed in the culture supernatants and the amount of released SVPs. Although the sedimentation peaks were not perfectly aligned, the intensity of the SVP peak decreased in the N154A mutant and increased in the D67N mutant (Fig 1G, right). These results suggest that the N-glycosylation of the E protein plays a crucial role in the release of SVPs.

### N-glycosylation of E protein is required for JEV proliferation

**Fig 2:**
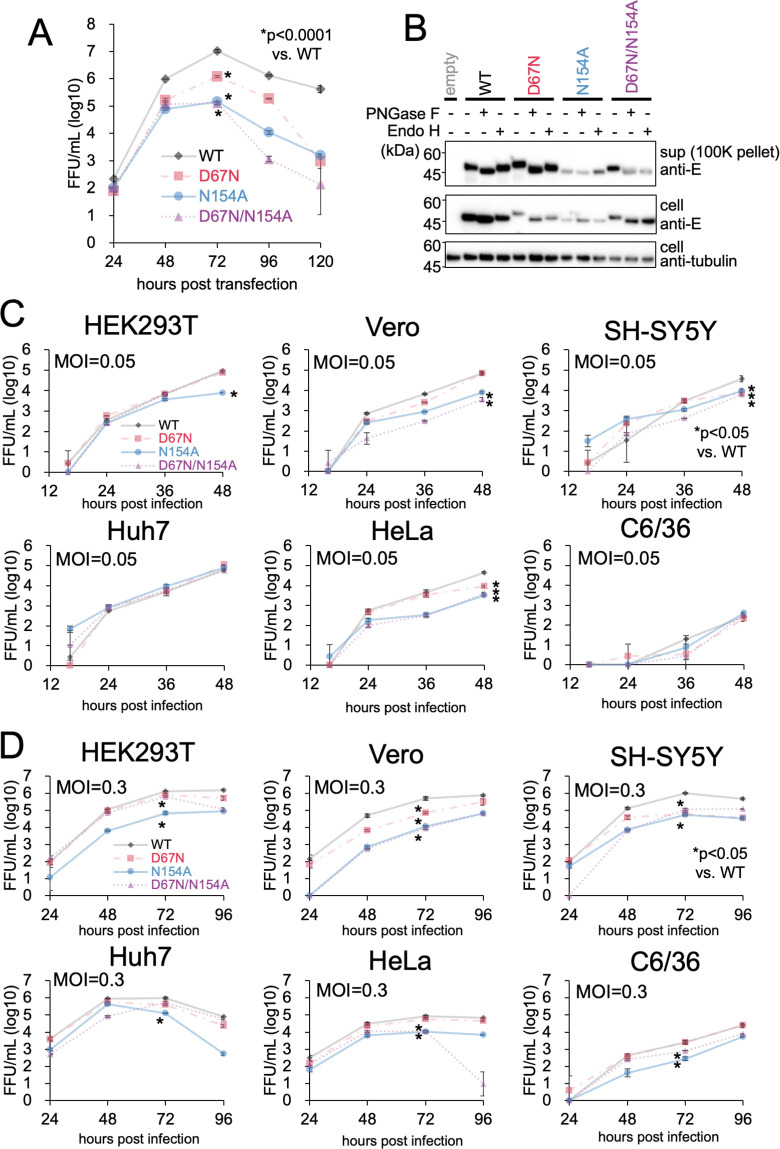
N-glycosylation of E protein is required for JEV proliferation in vitro. (A) Production of N-linked glycosylation (N-glycosylation) mutant JEV. 293T cells were transfected with a Circular Polymerase Extension Reaction (CPER) product that expressed infectious JEV RNA genome, supernatants were collected at the indicated time points, and infectious viral titers were determined. *p < 0.0001. (B) Immunoblotting analysis of E protein in cells transfected with JEV CPER products in (A). Cell lysates or supernatants obtained 5 days after transfection were subjected to PNGase F or Endo H treatment, followed by immunoblotting with anti-JEV E or anti-alpha-tubulin antibodies. (C and D) Growth kinetics of JEV mutants in different cell types. Cells were infected with various mutants at a multiplicity of infection (MOI) of 0.05 (C) or 0.3 (D), and the culture supernatants were collected at the indicated time points. The infectious titers were then determined by focus forming assays. *p < 0.05. Error bars represent the standard deviation. Means and SDs from three independent experiments are presented.

To examine the effect of N-glycosylation mutations on JEV growth, we produced recombinant JEV and evaluated their growth in cultured cells. Circular polymerase extension reaction (CPER) products [63,64]expressing JEV genomic RNA were transfected into 293T cells and the viral infectious titer in the culture supernatants was measured (Fig 2A). Immunoblotting revealed that the signals of the N154A mutant were lower than those of the WT, and the mobility of this band was almost identical to that of the WT E protein treated with Peptide N Glycosidase F (PNGase F) or Endoglycosidase H (Endo H) (Fig 2B). These results suggest that N154 is the only glycosylation site present in the JEV E proteins. After Endo H digestion, the WT E protein appears comparable to the E protein without glycosidase treatment, indicating that glycosylation at N154 remains unaffected by Endo H. In contrast, the D67N/N154A mutant exhibits altered mobility after Endo H digestion, exhibits similar mobility to the E protein after PNGase treatment, suggesting successful removal of the glycosylation at N67 (Fig 2B). These results suggest that the two asparagine residues at positions 67 and 154 may undergo glycosylation with different sugar compositions. The infectious titers of N154A and D67N/N154A were 100- and 10-fold lower than that of the WT, respectively, suggesting that N154 glycosylation is crucial for efficient virus propagation. Interestingly, the growth of the D67N mutant was also 3-fold lower than that of the WT, indicating that the presence of additional N-glycosylation negatively regulates virus propagation (Fig 2A).

To investigate the potential variations in the N-glycosylation dependence of virus propagation across different cell types, Huh7, Vero, SH-SY5Y, HeLa, C6/36, and 293T cells were subjected to secondary infection with N-glycosylation mutants. The resulting viral titers produced by each cell line were measured. In all tested cases, the growth of N-glycosylation mutants was lower than that of the WT, although the effect of the D67N mutation varied across cell types (Fig 2C and 2D). These findings suggest that N-glycosylation of the E protein is a common feature across various cell types, including insect cells. In the experiment conducted at an MOI of 0.05, the N154A mutation also influenced virus growth at an earlier time point, specifically at 48 h post-infection, in both Vero and HeLa cells (Fig 2C). This observation highlights the significance of N-glycosylation during the entry stage of the viral life cycle. These findings are consistent with a previous report indicating that the interaction between sugar chains on the E protein and cellular lectin proteins, such as DC-SIGN, is crucial for virus entry [33,54].

### Alteration of the end of the sugar chain is not critical for SVP secretion

**Fig 3:**
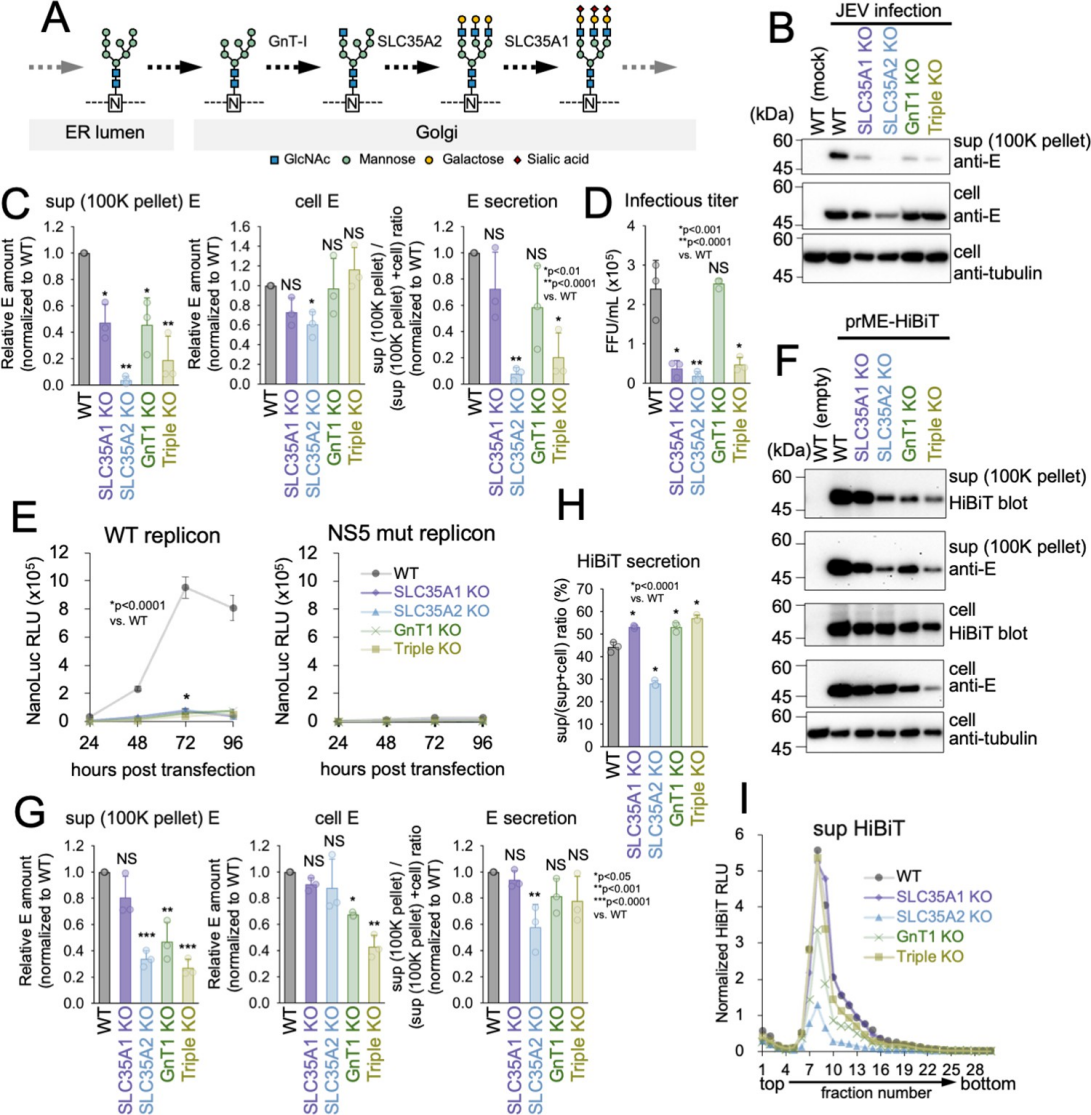
Modification of the terminal sugar chain in Golgi is not required for SVP secretion. (A) Schematic illustration showing the examined relevant factors (GnT-1, SLC35A2, and SLC35A1) in the Golgi involved in N-glycosylation pathways. (B) Detection of E protein in JEV-infected WT, SLC35A1 KO, SLC35A2 KO, GnT-1 KO, or Triple KO 293T cells. Cells were infected with JEV at MOI = 0.3 and harvested 48 hours post-infection. The amounts of E protein and alpha-tubulin in the cell lysate (cell) and in the 100,000 xg precipitate fraction of culture supernatant (100K pellet) were analyzed via immunoblotting using anti-E protein (top and second) and anti-alpha-tubulin antibodies (bottom), respectively. (C) Quantification of E protein in the 100K pellet (left) and cell (middle). Graphs show quantification of the band intensity from Immunoblots in (B). Secretion rate of E protein (right) were calculated by dividing the amount of the 100K pellet by the total E amount (100K pellet + cell). *p < 0.01; **p < 0.0001; NS, not significant. (D) JEV infectious titer in the culture supernatant of JEV-infected WT or KO 293T cells. Cells were infected with JEV at MOI = 0.3, and the culture supernatant was harvested 48 hours post-infection. Infectious titer in the culture supernatants was measured using focus forming assays. *p < 0.001; **p < 0.0001; NS, not significant. (E) Replication of the JEV subgenomic replicon in WT or KO 293T cells. Cells were transfected with a plasmid expressing JEV subgenomic replicon RNA that contains the NanoLuc (Nluc) luciferase reporter gene. Nluc luciferase activity in cells was monitored at the indicated time points (left panel). *p < 0.0001. Another experiment was performed using subgenomic replicon RNA containing a replication-defective mutation in the NS5 polymerase gene (NS5 mut replicon) as a negative control for the replicon RNA replication (right panel). (F) Detection of E protein in WT or KO 293T cells expressing JEV prME proteins. Cells were transfected with a prME-HiBiT-expressing vector and harvested 48 hours post-transfection. The amounts of E protein and alpha-tubulin in the cell lysate (cell) and in the 100,000 xg precipitate fraction of culture supernatant (100K pellet) were analyzed via immunoblotting using anti-E protein (second and forth) and anti-alpha-tubulin antibodies (bottom), respectively. E protein was also detected with HiBiT blotting (top and third panel). (G) Quantification of E protein in the 100K pellet (left) and cell (middle). Graphs show quantification of band intensity from the immunoblots using anti-E antibodies in (F). Secretion rates of E protein (right) were calculated by dividing the amount of the 100K pellet by the total E amount (100K pellet + cell). *p < 0.05; **p<0.001; ***p < 0.0001; NS, not significant. (H) HiBiT secretion rate of prME-HiBiT protein in WT or KO 293T cells. The HiBiT activities in both cells and 500 xg centrifugation fraction of culture supernatants in (F) were measured, and the secretion rate was calculated by dividing the HiBiT value in culture supernatants by the total HiBiT value in the cell culture (sup + cell). *p < 0.0001. (I) Sucrose density gradient fractionation of the SVPs released from WT or KO cells expressing the prME-HiBiT protein. Culture supernatants in (F) were subjected to 10–40% sucrose density gradient ultracentrifugation. HiBiT activities in each fraction were measured and normalized to cell E protein amount in (G). Error bars represent the standard deviation. Means and SDs from three independent experiments are presented.

Previous studies investigating the composition of N-linked glycosylated viral particles in TBEV and DENV have identified terminal sugar chains of galactose and sialic acid [40,41,65]. After conjugation and trimming in the ER, these N-glycans are transported to the Golgi apparatus where they undergo additional modifications by various glycosidases and glycosyltransferases. These modifications include the conjugation of galactose and sialic acid [39]. In the Golgi apparatus, SLC35A1 functions as a CMP-sialic acid transporter, SLC35A2 functions as a UDP-galactose translocator, and GnT-1 functions as an N-acetylglucosaminyltransferase. The deficiency of any of these enzymes limits the elongation of terminal glycans beyond that of sialic acid, galactose, and N-acetylglucosamine during N-linked glycosylation (Fig 3A). To investigate the significance of N-glycan modification, we examined JEV propagation and SVPs release in cells deficient in SLC35A1 (SLC35A1 KO), SLC35A2 (SLC35A2 KO), GnT-1 (GnT-1 KO), or all three (Triple KO). These cells were infected with JEV, and the cells and culture supernatants were collected 48 h post-infection to measure the quantity of released E protein and infectious titer. As shown in Fig 3B and 3C, the amount of E protein in culture supernatants was reduced in SLC35A1 KO, SLC35A2 KO, GnT1 KO, and Triple KO cells. The amount of E protein in cells was also reduced in SLC35A2 KO cells. The secretion rate of the E protein was determined by calculating the band intensities from immunoblotting, and it was found to be reduced in SLC35A2 KO and Triple KO cells (Fig 3C). The infection titer in the culture supernatants was also reduced in the SLC35A1 KO, SLC35A2 KO, or Triple KO cells (Fig 3D). These findings suggest that the modification of galactose and sialic acid is essential for JEV infection. To determine which stage of the viral life cycle was inhibited, its effect on viral genome replication was tested using a viral RNA replicon system. As shown in Fig 3E, the replication of the JEV genome was significantly reduced in cells lacking SLC35A1, SLC35A2, GnT-1, or all three genes, indicating that N-glycan modification mediated by these host factors is required for viral genome replication. In contrast, in SVPs secretion experiments, only a minor reduction in secretion was observed in SLC35A2 KO cells compared to WT (Fig 3F–3I). These reductions were also observed in GnT1 KO cells in sucrose density-purified SVPs (Fig 3I), suggesting that these N-glycan modifications were partially involved in SVPs release. Nonetheless, the extent of the decrease in SVPs observed in these KO cells was less pronounced than the effect of the N154A mutation, indicating that N-glycosylation may play a role in JEV particle release at different stages.

### Transport of N-glycosylation deficient E protein is arrested at ER

**Fig 4:**
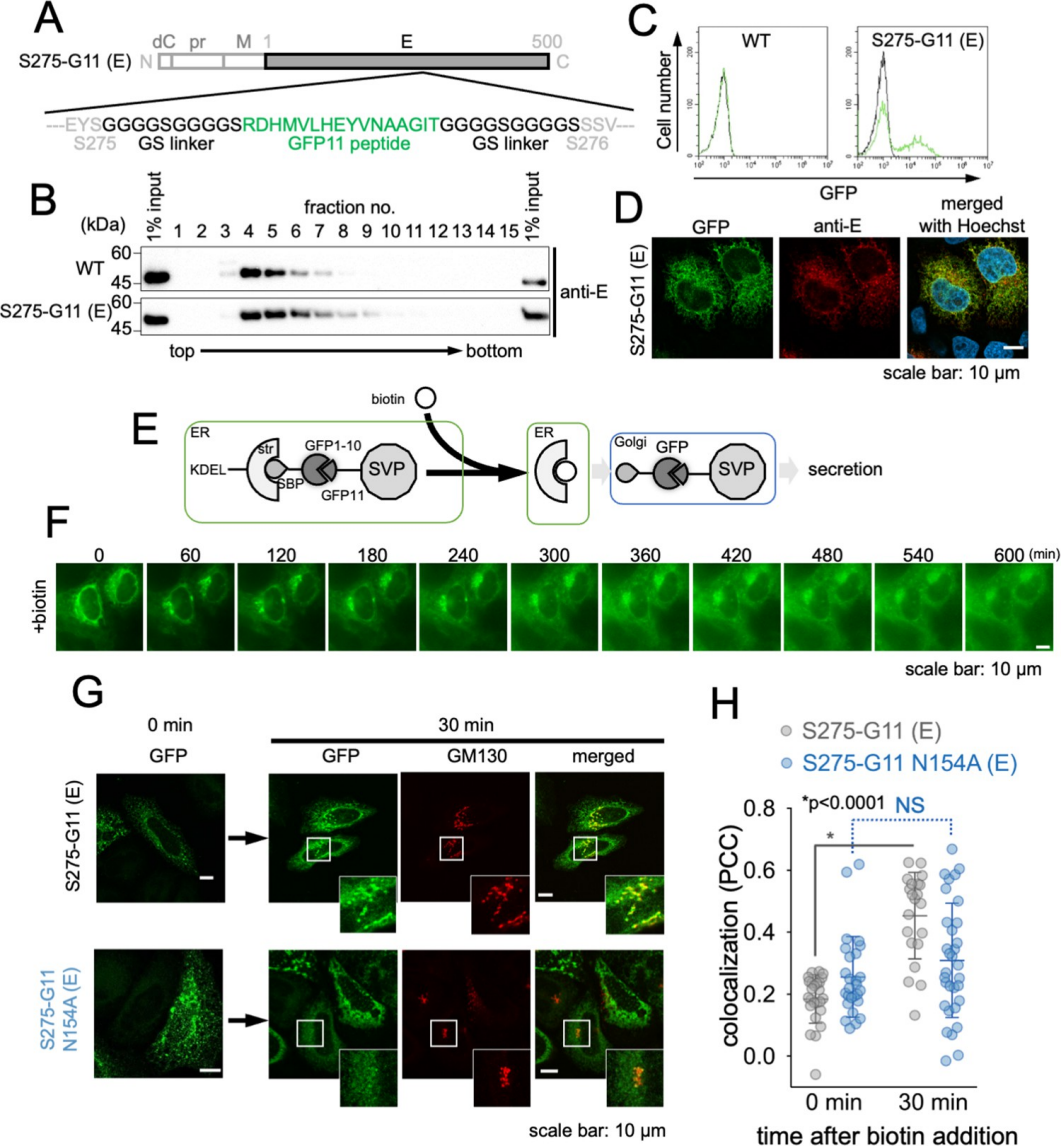
N-glycosylation-deficient E protein inhibits the ER-Golgi transition of SVPs. (A) Primary structure of a prME expression construct with GFP11 insertion (S275-G11 (E)). The GFP11 tag sequence (green) and GS linker sequence (black) are indicated. (B) Confirmation of SVP formation of GFP11-inserted prME protein. Cell lysates from the indicated construct-expressing 293T cells were fractionated using 10–40% sucrose density gradient ultracentrifugation and then E protein in each fraction was detected using immunoblotting with anti-E protein antibody. (C) GFP complementation in cells expressing prME (S275-G11 (E)) and GFP1-10. HeLa cells expressing GFP1-10 were transfected with prME WT (left panel) or prME (S275-G11 (E)) (right panel). GFP fluorescence of cells was analyzed using flow cytometry at 24 hours post-transfection. Black and green line in the panels indicates empty and prME vector transfected cell, respectively. (D) Subcellular localization of complemented GFP in cells expressing prME (S275-G11 (E)) and GFP1-10. HeLa cells expressing GFP1-10 were transfected with prME (S275-G11 (E)). At 24 hours post-transfection., cells were fixed and stained with anti-E antibody (red) and Hoechst (Cyan). GFP fluorescence (Green) was observed. Scale bar: 10 μm. (E) Outline of the strategy for the visualization of synchronized transport of SVPs by the RUSH system. In the absence of biotin, ss-Streptavidin-Binding Peptide (SBP) -GFP1-10 reporter with prME (S275-G11 (E)) is retained in the ER by the streptavidin-KDEL hook. After biotin addition, the reconstituted GFP-labeled SVPs leave the ER and translocate to the Golgi apparatus. (F) Timelapse imaging analysis of HeLa cells expressing GFP-labeled SVPs under the RUSH system. Images of GFP fluorescence are shown, and elapsed times after addition of biotin are indicated at the top of each panel. Scale bar: 10 μm. (G) Golgi transport of GFP-labeled SVPs. HeLa cells expressing RUSH-GFP-SVPs WT (top panels) or N154A (bottom panels) were fixed and stained with anti-GM130 (red) at 0 min (left panels) or 30 min (right panels) after biotin addition, and GFP fluorescence (green) was observed. Scale bar: 10 μm. (H) Colocalization of GFP and GM130 signals (G) was determined by calculating Pearson’s correlation coefficient. *p < 0.0001; NS, not significant. Error bars represent standard deviation. Means and SDs from three independent experiments are presented.

To identify the intracellular trafficking process responsible for the impaired secretion of N-glycan-deficient SVPs, we performed live cell imaging. To enable fluorescence visualization of intracellular SVPs, we utilized a prME construct containing a GFP-11 peptide (S275-G11), a part of the split green fluorescent protein (GFP), which was inserted into the tolerance site of the E protein, as previously described [66](Fig 4A). Sucrose density gradient centrifugation was performed to analyze cell lysates from cells expressing either WT or S275-G11 prME. The analysis revealed that prME (S275-G11) co-sedimented with WT prME in the same fraction (Fig 4B), suggesting that prME (S275-G11) forms SVPs in the cells. GFP fluorescence was also observed when prME S275-G11 was co-expressed with GFP1-10, another part of the split GFP, in HeLa cells (Fig 4C), and GFP fluorescence and immune-stained E protein were co-localized in the ER (Fig 4D). These results indicated that the SVPs were fluorescently labeled with GFP in the cells. To track the intracellular dynamics of SVPs, we utilized the retention using selective hook (RUSH) system. The RUSH system enabled synchronization of GFP-labeled SVP secretion by capturing it in the ER lumen via KDEL-hook binding and regulating its simultaneous release from the ER through the addition of biotin (Fig 4E) [67]. The addition of biotin to cells co-expressing the prME S275-G11 and RUSH constructs resulted in the migration of GFP fluorescence from the ER to the Golgi and subsequently from the Golgi to the endosome (Fig 4F), suggesting that this system visualizes the transport process of GFP-labeled prME (which corresponds to SVPs) from the ER, through the Golgi, and finally to the endosome. Next, the N154A prME transport was tracked using this system. 30 min after the addition of biotin, the cells were fixed, and the migration of GFP fluorescence to the Golgi was evaluated by staining with an antibody against GM130, a cis-Golgi marker. The limited co-localization of prME N154A with GM130, as compared to prME WT, indicated that the transport of the N154A mutant was arrested in the ER (Fig 4G and 4H). These results suggest that N154 glycosylation of the E protein is necessary for its transport from the ER to the Golgi apparatus.

### Host sugar-chain recognizing cargo receptors, ERGIC-53/ERGIC-L, are not required for export of E from ER

**Fig 5:**
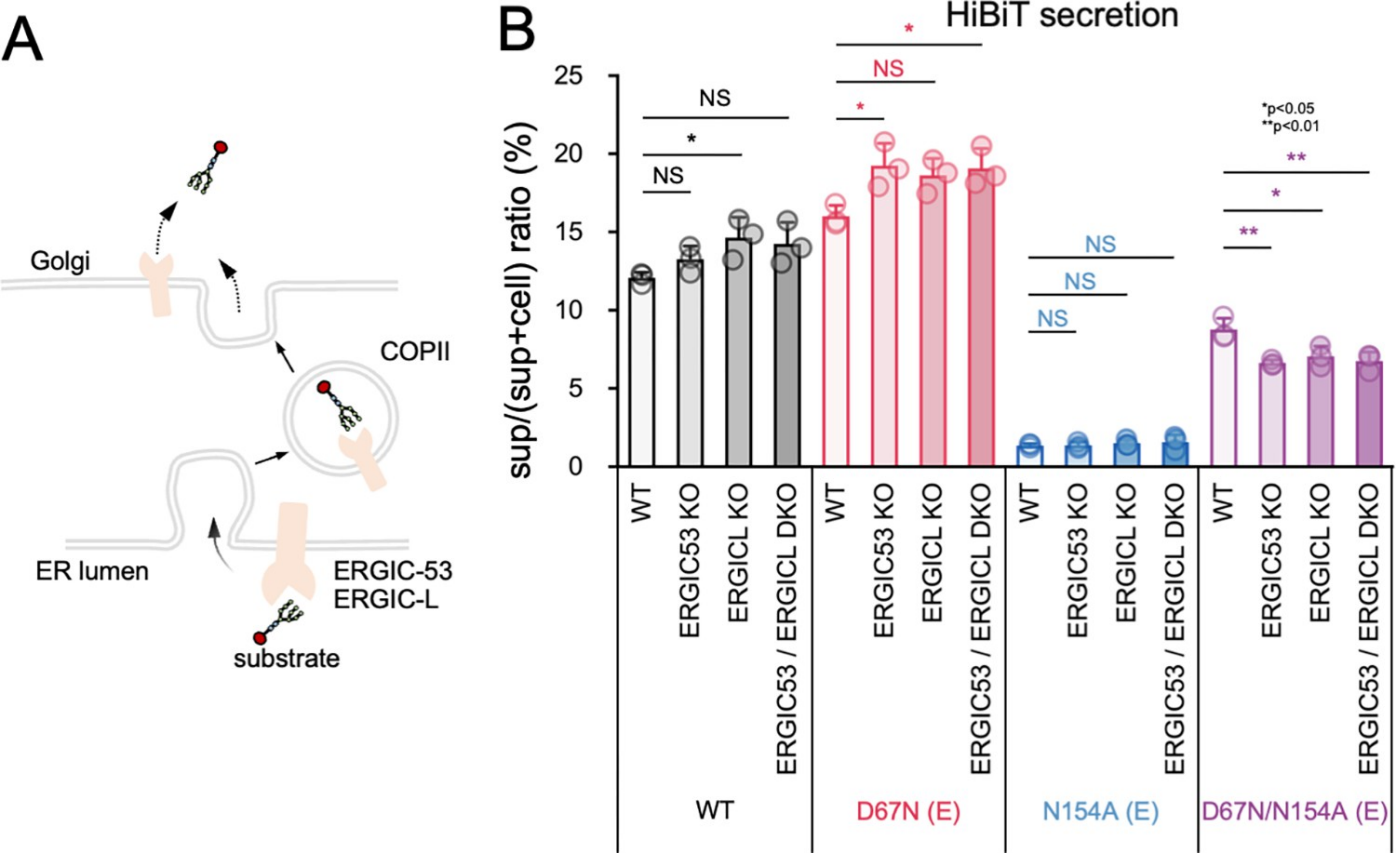
Cargo receptors ERGIC-53 and ERGIC-L, which recognize sugar chains, are not necessary for the transport of E protein from the ER to the Golgi. (A) Illustration of the anterograde transport of glycosylated cargo proteins by ERGIC-53 or ERGIC-L. (B) Effect of ERGIC-53 and/or ERGIC-L deletion on the secretion of SVPs. WT, ERGIC-53 KO, ERGIC-L KO, or ERGIC-53/ERGIC-L double KO (DKO) HCT116 cells were transfected with the indicated prME-expressing constructs. Cells and culture supernatants were harvested 48 hours-post transfection, and the corresponding HiBiT activities were quantified. Secretion rates were determined by dividing the HiBiT value in culture supernatants by the total HiBiT value in the cell culture. *p < 0.05; **p<0.01; NS, not significant. Error bars indicate the standard deviation. Means and SDs from three independent experiments are presented.

ERGIC53 and ERGIC-L are cargo receptors responsible for glycan recognition and COPII transport from the ER to the Golgi apparatus (Fig 5A) [68]. To investigate whether ERGIC53 and ERGIC-L recognize E-glycosylation, we tested the secretion of SVP-HiBiT in cells lacking ERGIC53, ERGIC-L, or both. The absence of ERGIC53, ERGIC-L, or both had no significant effect on SVP secretion (Fig 5B). These results suggest that ERGIC53 and ERGIC-L have a negligible effect on the secretion of viral particles.

### Deficiency of N-glycosylation impairs solubilization of E protein and cause of rapid degradation by host ER associated degradation (ERAD) system

**Fig 6:**
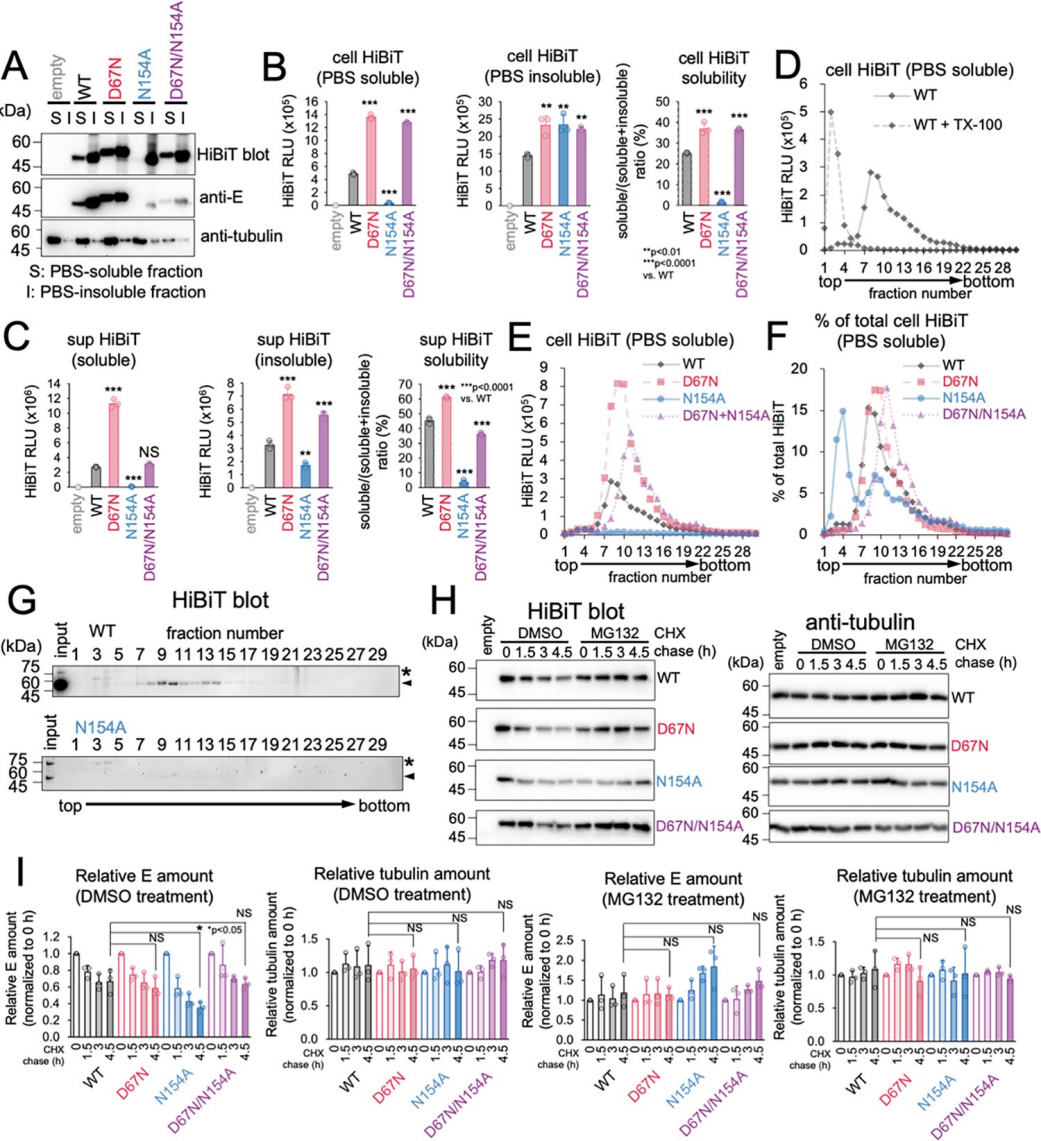
E protein N-glycosylation deficiency impairs its solubilization and causes rapid degradation by the host ERAD system. (A) Immunoblotting analysis of E protein solubility in the cells. 293T cells were transfected with the indicated prME-HiBiT-expressing constructs, and at 48 hours post-transfection the cell suspended in the PBS buffer underwent four cycles of freeze-thawing. Subsequently, the soluble (S) and insoluble (I) fractions were separated via centrifugation, and HiBiT-blotting (top panel) and immunoblotting using anti-JEV E protein (second panel) or anti-alpha tubulin (third panel) antibodies were performed to detect these proteins in the S and I fractions. (B and C) HiBiT activity measurement analysis of E protein solubility. HiBiT activities in the S and I fractions prepared from the cells (B) or supernatant (C) were measured. Solubility was calculated by dividing the HiBiT value in the S fraction by the total HiBiT value of the S and I fractions. Statistical significance was observed with **p < 0.01 and ***p < 0.0001. (D) Detection of SVPs in the S fraction derived from cell lysates by sucrose density gradient analysis. Soluble fraction of the cell lysates was pretreated with (dotted line) or without (solid line) 1% Triton X-100 and subjected to 10–40% sucrose density gradient ultracentrifugation. HiBiT activities in each fraction were measured. (E) The amount of SVPs changed in the S fraction of cells expressing each prME-HiBiT protein. The S fraction from 293T cells expressing WT or mutant prME-HiBiT was subjected to 10–40% sucrose density gradient ultracentrifugation. HiBiT activities in each fraction were measured. (F) Normalized representation of the amount of prME-HiBiT protein in each fraction. The Y-axis displays the percentage of HiBiT activity detected in each fraction in (E). (G) HiBiT-blotting analysis of sucrose density gradient-purified prME-HiBiT proteins. S fraction derived from WT (top panel) or N154A mutant (bottom panel) prME-HiBiT protein-expressing 293T cells was subjected to 10–40% sucrose density gradient ultracentrifugation. E protein in each fraction was detected using HiBiT blotting. (H) Stability of E protein. Cycloheximide chase analysis was performed to evaluate the stability of E protein. The plasmids expressing WT or mutant prME-HiBiT proteins were transfected into 293T cells. After 48 hours post-transfection, 50 μg/ml of cycloheximide with dimethyl sulfoxide (DMSO) (as a negative control) or 10 μM MG132 were added to the cell culture, and samples were collected at the indicated time points after cycloheximide treatment. The E protein (left panels) or alpha-tubulin (right panels) in the samples were detected using HiBiT-blotting or immunoblotting with an anti-alpha-tubulin antibody. (I) Quantification of band intensities in (H). Band intensities in (H) were measured. *p < 0.05; NS, not significant. Standard deviation is represented by error bars. Means and SDs from three independent experiments are presented.

Protein folding in the ER lumen is regulated by glycosylation, which ensures that only correctly folded glycoproteins are transported to the Golgi apparatus [69]. The solubility of this mutant in the ER membrane was tested to determine the reason for the arrest of prME N154A transport in the ER. Cell or culture supernatant fractions from the cells expressing E proteins were subjected to a freeze-thaw cycle in phosphate-buffered saline (PBS) and the solubility of E protein were compared between WT and mutants. After centrifugation, the supernatant (soluble fraction) and pellets (insoluble fraction) were evaluated for E protein content using immunoblot analysis (Fig 6A) or HiBiT activity measurements (Fig 6B). The results indicated that the D67N and D67N/N154A mutations led to a 1.4-fold increase in solubility compared to the wild type, whereas the N154A mutation caused a significant decrease of approximately 14-fold in solubility (Fig 6B). Similar results were also obtained from the culture supernatant fraction (Fig 6C). These results suggest that the degree of glycosylation plays a significant role in the solubilization of E proteins. A single HiBiT sedimentation peak was observed during the sucrose density gradient centrifugation of the soluble cell lysate from prME-expressing cells. However, pretreatment with the detergent caused this peak to disappear (Fig 6D). These findings strongly suggested that the HiBiT peak fractions in this experiment contained assembled SVPs within the cell. The observed sedimentation peak was elevated in the case of the D67N mutation, whereas it was not discernible in the N154A mutation (Fig 6E), suggesting that N-glycosylation plays a critical role in the assembly of SVPs in cells. Magnification of the y-axis of the graph depicted in Fig 6E revealed additional peaks (Nos. 3–5) in the N154A mutant in the lighter sedimentation region (Fig 6F). HiBiT blot analysis indicated the presence of bands corresponding to unprocessed prME in these fractions (Fig 6G), implying that N-glycosylation is indispensable for proper processing of prME as well as assembly of SVPs. Misfolded proteins in the ER lumen are eventually retrogradely transported to the cytoplasm, where they are degraded by the ubiquitin-proteasome system. This mechanism is referred to as the ERAD [70]. To assess the stability of the prME WT and N-glycosylation mutants, cycloheximide (CHX) chase analysis was performed (Fig 6H and 6I). The results showed that the N154A mutant exhibited a more rapid degradation rate than the wild type, and its degradation was inhibited by the proteasome inhibitor MG132. These results suggested that N154A is primarily subjected to substrate recognition and degradation by ERAD. Hence, it can be inferred that N-glycosylation may play a crucial role in stabilizing prME and SVPs by facilitating their proper folding.

### The secretion and solubility rates of E protein from other natural isolates of flaviviruses that lack the N-glycosylation site

**Fig 7:**
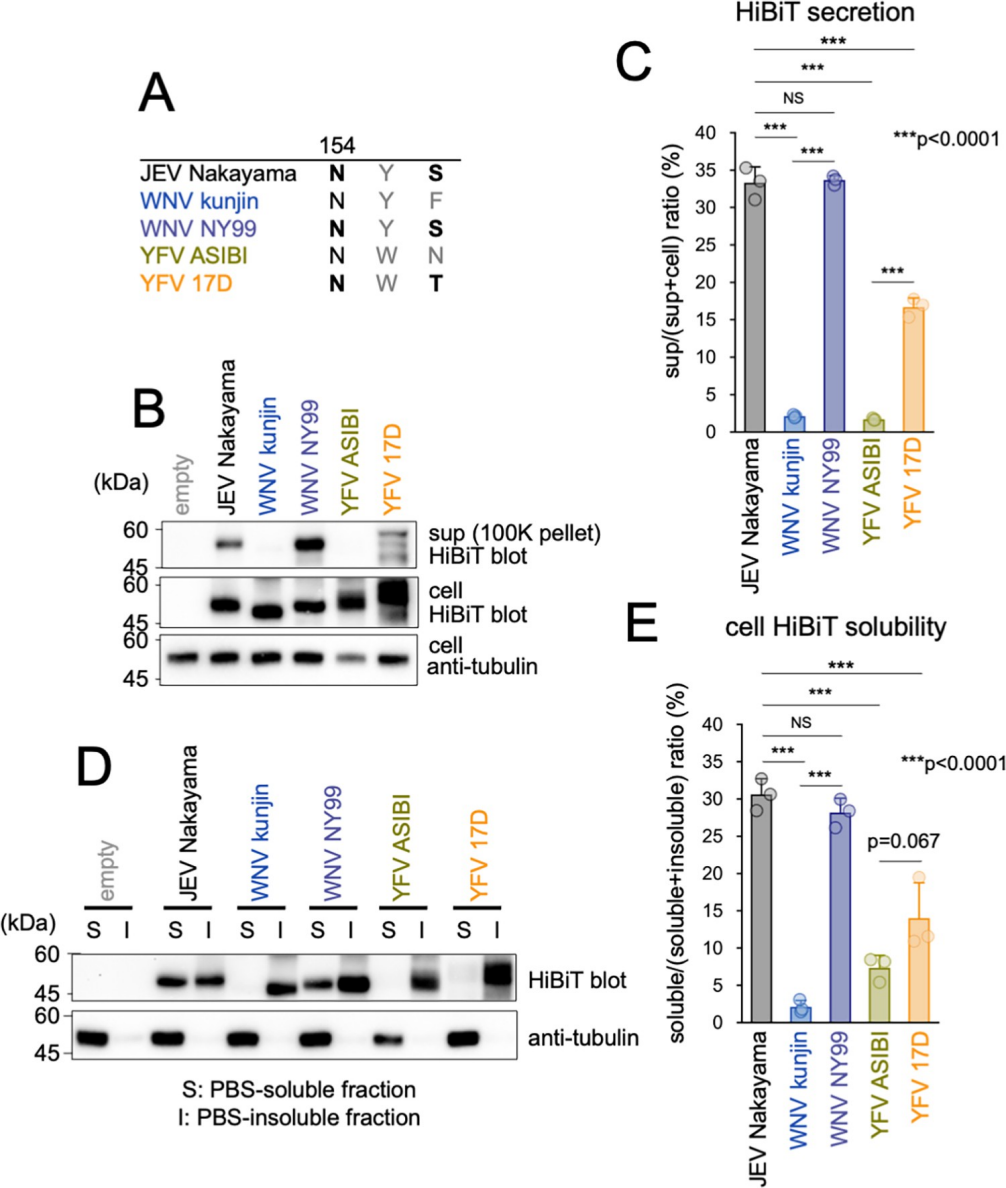
Secretion and solubility rates of E protein of other flavivirus natural isolates. (A) Amino acid sequence spanning residues 154 to 156 in the E protein of JEV, WNV, or YFV. The sequences are derived from JEV Nakayama [83], WNV NY99 (GenBank accession no. DQ211652.1), WNV kunjin (KX394384.1), YFV 17D (JX949181), and YFV ASIBI (AY640589). (B) Immunoblotting analysis of SVP release. 293T cells transfected with the indicated prME-HiBiT constructs were cultured for 48 h, and then cells and culture supernatant were harvested. The amounts of E protein (middle panel) and alpha-tubulin (bottom panel) in cell lysates and E protein in the100K pellet fraction of culture supernatant (top panel) were analyzed using HiBiT blotting or immunoblotting with an anti-alpha-tubulin antibody, respectively. (C) HiBiT activity measurement analysis of SVP release. HiBiT activity in cell lysates and 500 xg centrifugation fraction of culture supernatants in (B) was measured and secretion rates were calculated. Standard deviation is represented by error bars. ***p < 0.0001; NS, not significant. (D) Immunoblotting analysis of E protein solubility. 293T cells were transfected with the indicated prME-HiBiT-expressing constructs, and at 48 hours post-transfection the cells underwent four cycles of freeze-thawing. Subsequently, the soluble (S) and insoluble (I) fractions were separated via centrifugation, and HiBiT-blotting and immunoblotting using an anti-alpha tubulin antibody were performed. (E) HiBiT activity measurement analysis of E protein solubility. HiBiT activities in each fraction in (D) were measured. Solubility was calculated by dividing the HiBiT value in the S fraction by the total HiBiT value of the S and I fractions. ***p < 0.0001; NS, not significant. Error bars represent standard deviation. Means and SDs from three independent experiments are presented.

Certain viral species classified under the Flavivirus genus comprise strains that do not possess a conserved N-glycosylation site in the E protein [32]. According to previous reports, the NY99 WNV strain undergoes N-glycosylation, whereas the Kunjin strain does not [43,44] (Fig 7A). Similarly, it has been observed that the 17D strain of YFV is N-glycosylated, whereas the ASIBI strain is not [46] (Fig 7A). We tested the secretion rates of SVPs derived from these naturally isolated flaviviruses. Immunoblotting analysis (Fig 7B) and the HiBiT assay (Fig 7C) revealed a significantly lower secretion rate of WNV Kunjin or YFV ASIBI, which are non-glycosylated strains, compared to that of WNV NY99 or YFV 17D, which are N154 glycosylated strains. Furthermore, the solubility of WNV Kunjin and YFV ASIBI prME was lower than that of WNV NY99 and YFV 17D (Fig 7D and 7E). These results suggest that N-glycosylation of the N154 residue plays a critical role in the proper folding of both WNV and YFV SVPs.

### The lack of N-glycosylation in the prM protein also negatively affects the solubilization of prM-E

**Fig 8:**
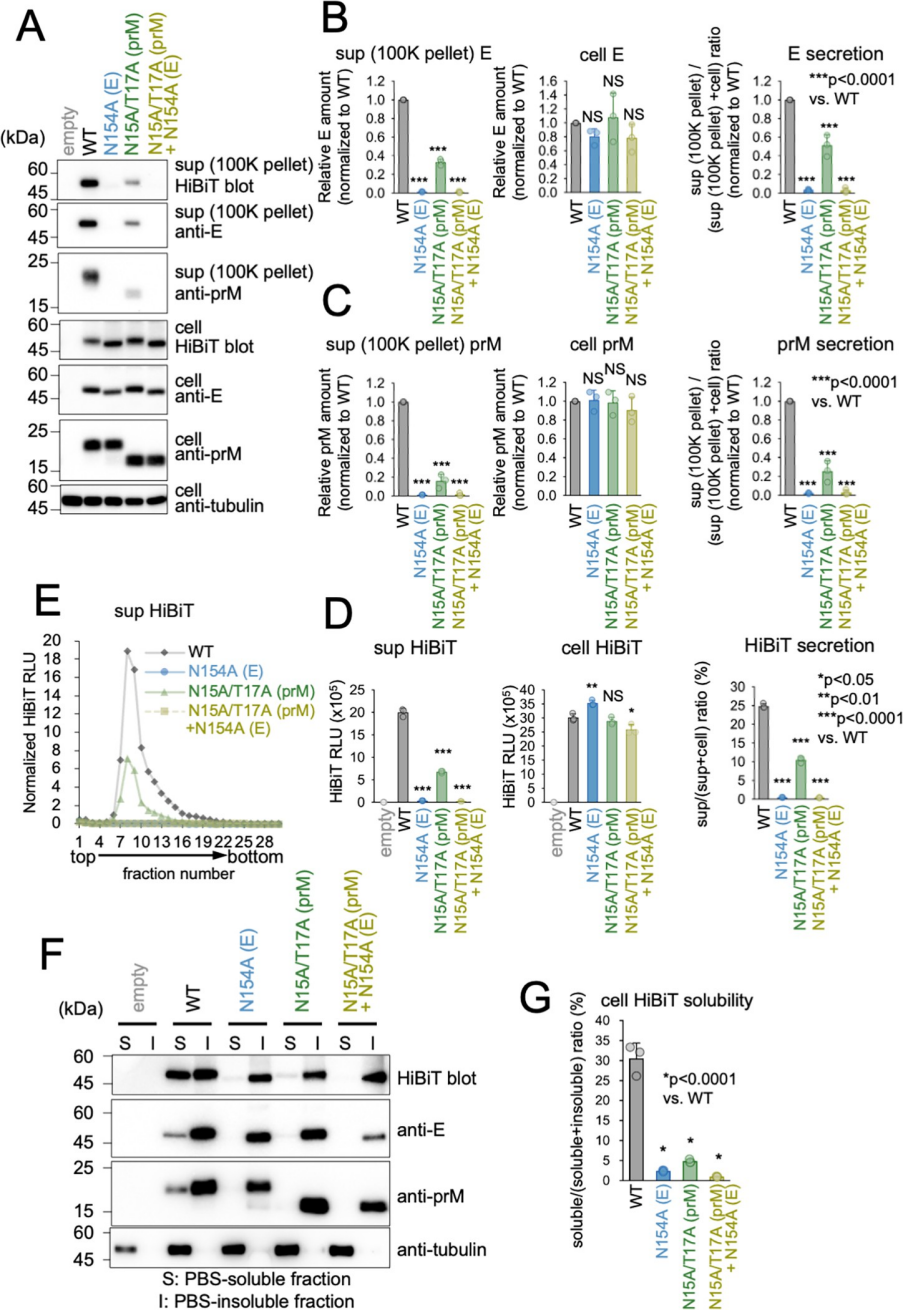
prM N-glycosylation deficiency impairs solubilization of prME. (A) Immunoblotting analysis of WT or mutant SVPs release. 293T cells transfected with the indicated prME-HiBiT constructs were cultured for 48 h, and then cells and culture supernatant were harvested. The amounts of E protein (fourth and fifth panel), prM protein (sixth panel), and alpha-tubulin (bottom panel) in cell lysates and the amounts of E protein (top and second panel) and prM protein (third panel) in the 100K pellet fraction of culture supernatants were analyzed using HiBiT blotting or immunoblotting with anti-E protein, anti-prM protein, or anti-alpha-tubulin antibodies. (B and C) Quantification of E (B) or prM (C) proteins in the 100K pellet (left) and cell (middle). Graphs show quantification of the immunoblots in (A). Secretion rates of E and prM proteins (right) were calculated by dividing the amount of the 100K pellet by the total E and prM amount (100K pellet + cell), respectively. ***p < 0.0001; NS, not significant. (D) HiBiT activity measurement analysis of WT or mutant SVP release. HiBiT activities in cell lysates (middle panel) and 500 × g centrifugation fraction of culture supernatants (left panel) in (A) were measured and secretion rates (right panel) calculated. Standard deviation is represented by error bars. *p < 0.05; **p < 0.01; ***p < 0.0001; NS, not significant. (E) Sucrose density gradient fractionation analysis of released WT or mutant SVPs. Culture supernatants of 293T cells expressing WT or mutant prME-HiBiT proteins were subjected to 10–40% sucrose density gradient ultracentrifugation. HiBiT activities in each fraction were measured and normalized to cell prM protein amount in (C). (F) Immunoblotting analysis of E protein and prM protein solubility. 293T cells were transfected with the indicated prME-HiBiT-expressing constructs, and at 48 hours post-transfection the cells underwent four cycles of freeze-thawing. Subsequently, the soluble (S) and insoluble (I) fractions were separated via centrifugation, and HiBiT-blotting (top panel) and immunoblotting using anti-E protein (second panel), anti-prM protein (third panel), or anti-alpha tubulin (bottom panel) antibodies were performed. (G) HiBiT activity measurement analysis of E protein solubility. HiBiT activities in each fraction in (F) were measured. Solubility was calculated by dividing the HiBiT value in the S fraction by the total HiBiT value of the S and I fractions. *p < 0.0001. Error bars represent standard deviation. Means and SDs from three independent experiments are presented.

Given that prM also undergoes N-glycosylation, we examined the effect of JEV prM N-glycosylation on viral particle secretion. A single N-glycosylation site (N15) has been identified in the prM region of JEV. Immunoblotting analysis and the HiBiT measurement assay revealed that the N15A/T17A mutant exhibited an approximately 2-fold decrease in prME secretion compared to the WT (Fig 8A–8D). Additionally, sucrose density gradient analysis also revealed a similar 2-fold decrease in the SVP HiBiT peak (Fig 8E). These results indicated that N-glycosylation of prM also plays a role in the efficient release of SVPs. Similarly, the solubility of prM in cells expressing the N15A/T17A mutant was significantly reduced compared to that in the WT (Fig 8F and 8G), indicating that N-glycosylation of prM is critical for the proper folding of prME and assembly of SVPs.

## Discussion

**Fig 9:**
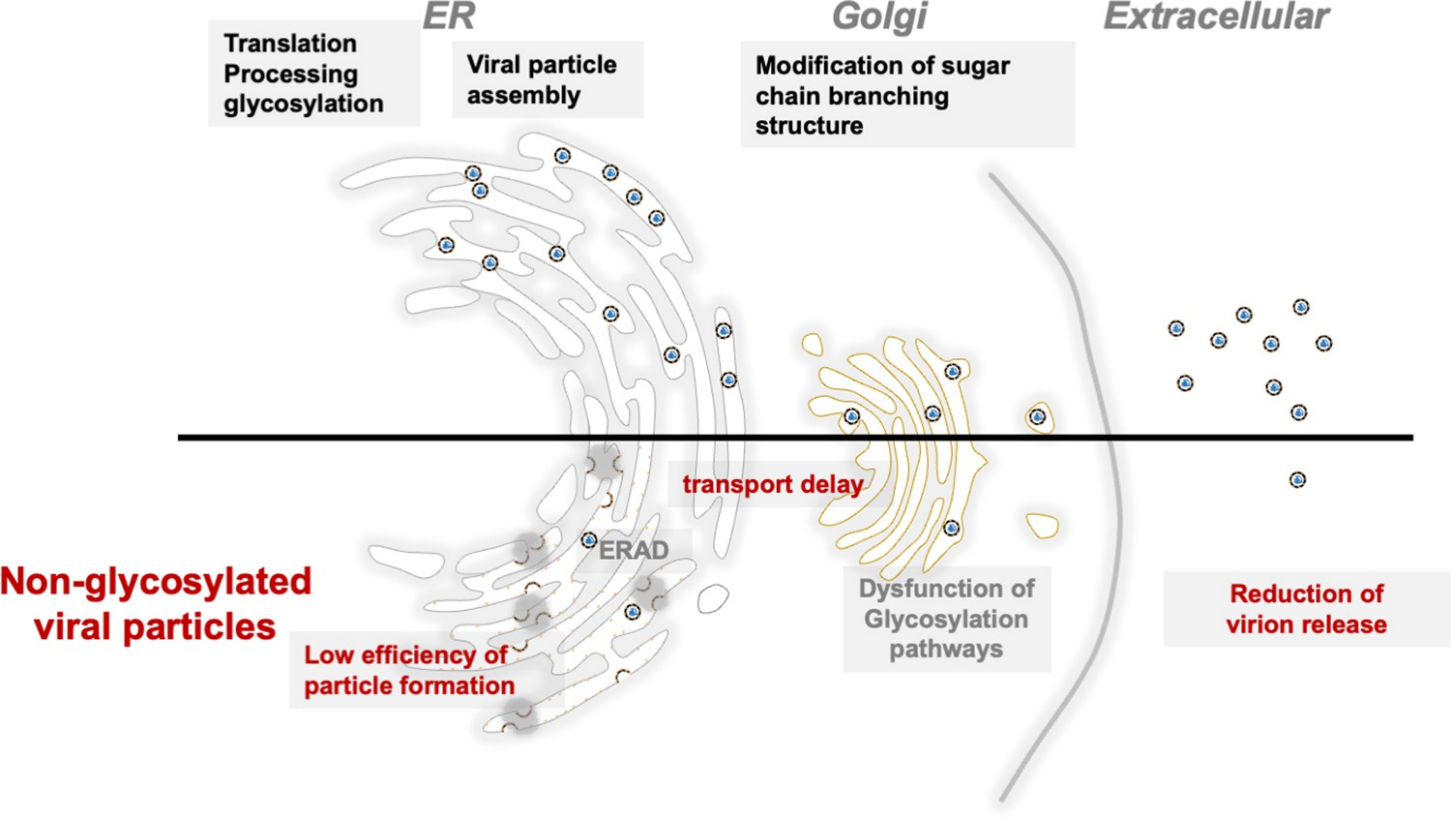
Model of the roles of prME N-glycosylation in viral particle formation and release. Mutations at the N-glycosylation site of both prM and E result in the accumulation of unprocessed and misfolded prME, preventing the assembly of virions or SVPs in the ER. This leads to delay in the transport of viral particles from the ER to the Golgi, eventually reducing the secretion of viral particles.

N-glycosylation of flavivirus E proteins has been linked to their ability to bind to target cells and achieve cell directionality. N-Glycosylation of the E protein in WNV and Murray Valley encephalitis virus (MVEV) leads to increased toxicity and the development of encephalitis [51,52]. Furthermore, a previous study on JEV, utilizing a mutant virus similar to that used in the current study, found that N154A substitution decreased mouse neurotoxicity and neuroinvasiveness [33]. In addition to this function, this study provides evidence that N-glycosylation of the E and prM proteins in flaviviruses plays a significant role in viral particle secretion by facilitating proper folding and solubilization of the prME protein and promoting its assembly. The absence of N-glycosylation slows the transition of SVPs from the ER to the Golgi apparatus, subsequently leading to reduced particle secretion (Fig 9).

The folding of proteins with N-glycans in the ER lumen is regulated by the state of the glycans attached to the glycosylation site [69]. ER chaperones such as calnexin and calreticulin recognize the transfer of sugar chains (consisting of glucose, mannose, and N-acetylglucosamine) to the N-glycosylation sites of nascent peptides, enabling proper protein folding. During this process, glucosidase removes glucose, and the modified glycoprotein is transported to the Golgi apparatus. Our study revealed that the absence of a glycosylation site impairs the folding of the prME protein as well as the assembly and transport of viral particles from the ER to the Golgi, highlighting the essential role of N-glycosylation in these processes.

The N154A mutation in the E protein significantly reduced the production of SVPs (Fig 1G), but its impact on JEV growth was not substantial (Fig 2A, 2C and 2D). This suggests that the mechanisms underlying SVP formation and formation of infectious viral particles are similar but different. SVPs do not contain capsids or viral genomic RNAs, which could potentially affect the prME assembly pattern. Previous reports indicated that SVPs consist of 60 copies of E packed as dimers in a T = 1 icosahedral structure [15,71]. N-Glycosylation may affect the formation of SVPs or infectious particles in different ways.

The infection titer of the D67N mutant was lower than that of the WT, and there was a significant increase in SVP release in the D67N mutant (Figs 1C and Fig 2B). This discrepancy could be explained by the possibility that additional N-glycan conjugation at this site negatively regulates viral replication at another step, such as viral entry. A previous study has demonstrated that EDII plays a crucial role in the fusion of the viral membrane with the endosomal membrane of target cells [20–22]. The D67N mutation may also impact the incorporation rate of the prM protein into virions. Immunoblotting analysis revealed higher levels of prM protein in the 100 K pellet fraction of the culture supernatants for the D67N and D67N/N154A mutants than for the WT (Fig 1C and 1E). Furthermore, the sedimentation peaks of the D67N and D67N/N154A mutants were observed at higher fractions than those of the wild-type (Fig 1G). These findings suggest that the D67N mutation leads to the preferential incorporation of the prM protein into virions. A previous study reported that prM is involved in stabilizing the E dimer [24]. Additional glycosylation may affect the interaction between prM and E and the normal maturation of SVPs. This study revealed distinct sugar compositions at two asparagine residues, N67 and N154. Glycosidase treatment revealed that the WT E protein, with glycosylation at N154, showed resistance to Endo H treatment, whereas the D67N/N154A mutant displayed sensitivity to Endo H treatment (Fig 2B). These findings suggest that the N67 and N154 can be glycosylated with different sugar compositions. Interestingly, these results align with prior reports indicating that residues at position 67 are modified by simple mannose glycans, whereas residues at position 154 are modified by complex glycans [33,48]. The presence of high-mannose glycans at N67 may play an inhibitory role against viral infections. These, along with other reasons, could explain why this N-glycosylation site is not conserved in flaviviruses, except in DENV.

In addition, this study revealed that modification of the terminal sugar chain in the Golgi apparatus is implicated in particle release. This was supported by the observation that SVPs release was partially inhibited in cells lacking either SLC35A12 or GnT-1 (Fig 3E–3G). These modifications might contribute to the stability of the assembled SVPs as well as other unknown host factors that could potentially be involved in the membrane transport of SVPs within cells. Interestingly, deficiency in SLC35A1, SLC35A2, or GnT-1 caused a significant impairment in viral genome replication (Fig 3B–3E). The findings of this study may be linked to those of a previous investigation that identified N-glycan synthesis-related genes through genome-wide screening of flavivirus replication. The absence of the oligosaccharyltransferase (OST) complex, which is responsible for transferring oligosaccharides to the N-linked glycosylation sites of nascent polypeptide chains, causes defects in DENV, YFV, and ZIKV genome replication [72–74]. This could be explained by the insufficient N-glycan modification of other viral proteins or unknown host factors involved in viral genome replication. It has been reported that the flaviviral non-structural proteins NS1 and NS4B undergo N-glycosylation [75]. Studies have shown that mutations in the NS1 or NS4B N-glycosylation sites in DENV can impair viral genome replication [76,77]. Although N-glycosylation of JEV NS1 and NS4B has not been extensively studied, it is possible that N-glycan modification of these NS proteins could influence JEV replication in cells.

In this study, we successfully visualized and tracked the transport of SVPs from the ER to the endosomes using the RUSH system (Fig 4A–4F). This time-lapse imaging study confirmed that viral particle release was facilitated by the conventional secretion pathway. The sorting of glycosylated cargo proteins is facilitated by cargo sorting receptors, such as ERGIC-53 and ERGIC-L, which specifically recognize N-glycans. Nonetheless, our study revealed that sorting of SVPs was not mediated by ERGIC-53 or ERGIC-L (Fig 5). These findings are not contradictory to those of previous studies indicating the involvement of the KDEL receptor, another cargo-sorting receptor, in the transportation of flavivirus particles from the ER to the Golgi [10]. It is possible that N-glycosylation of viral particles does not play a significant role in this step.

The number and location of predicted N-glycosylation sites in the E protein are not conserved among flaviviruses [32,42]. Our study demonstrated that the N-glycosylated strains of both WNV and YFV showed improved secretion and higher intracellular solubility than the non-glycosylated strains (Fig 7). This finding is consistent with previous studies demonstrating the impact of the absence of an E protein glycosylation site in the WNV NY99 strain on the secretion of SVPs [58]. Moreover, research has indicated that N-glycosylation of the E protein in the WNV confers an advantage in terms of growth in mosquito cells [53] and pathogenesis in mice [51]. Although it has been suggested that this advantage may be a result of enhanced attachment to target cells [33,54,78–81], it is also possible that this is due to increased efficiency in particle secretion. Notably, non-glycosylated strains of flaviviruses with E proteins have been isolated from nature. This suggests that N-glycosylation of the E protein may not always be advantageous for the survival of flaviviruses in natural environments.

In this study, we explored the role of JEV particle surface glycosylation in JEV release. Our study demonstrates that N-glycosylation of particles plays a critical role in particle assembly, intracellular transport, and infectivity. Targeting N-glycosylation during drug discovery can potentially modify the properties of viruses and produce antiviral effects.

## Materials and methods

### Cells, viruses, and transfection

The 293A, Vero, HeLa, Huh7, SH-SY5Y, 293T-derived knockout cell lines [82], and HCT116-derived knockout cell lines [68] were cultured in Dulbecco’s modified Eagle medium (DMEM)-high glucose (Nacalai Tesque, Kyoto, Japan) containing 100 units/mL penicillin, 100 μg/mL streptomycin, and 10% (v/v) fetal calf serum (FCS), in a humidified atmosphere containing 5% CO_2_ at 37° C. C6/36 cells were cultured in Schneider’s insect medium (Merck KGaA, Darmstadt, Germany) containing 100 units/mL penicillin, 100 μg/mL streptomycin, and 10% (v/v) FCS at 25°C. The JEV AT31 strain (kindly provided by Eiji Konishi, Osaka University, Japan) was propagated in 293A cells. PEI MAX (Polysciences, Warrington, PA, USA) or Lipofectamine 3000 (Thermo Fisher Scientific, Waltham, MA, USA) were used for transfection according to the manufacturer’s protocols.

### Plasmid construction

All constructs employed in this study are summarized in Table 1, and the pCAG-JEprME vector was kindly donated by Ryosuke Suzuki [83]. The pCAG-JEprME-HiBiT construct was created by inserting the prME coding sequence tagged at the C-terminus with a HiBiT tag, which was amplified using pCAG-JEprME [83] as a template, into the KpnI/XhoI site of pCAG-MCS2 [84]. Mutant variants of the JEV prME sequences, including alanine, asparagine substitutions or GFP11 tag-inserted forms, were generated by overlap extension polymerase chain reaction (PCR) and subsequently cloned into the same site in pCAG-MCS2. The coding sequence of prME was tagged at the C-terminus with the HiBiT tag from WNV NY99 (GenBank accession No. DQ211652.1), WNV Kunjin (KX394384.1), YFV 17D (JX949181), and YFV ASIBI (AY640589) were synthesized by Twist Bioscience (San Francisco, CA, USA), and cloned into the same site as pCAG-MCS2. The wildtype and RdRp-inactivated subgenomic reporter replicon-expressing plasmids, pCMV (Δ4.5p)-JErep-nluc and pCMV (Δ4.5p)-JErep-nluc-fs, respectively, were previously described [85].

**Table 1:** Plasmid list.

Plasmid Name	Backbone	Selection	Cloning Sites	Source
pCAG-MCS2	-	Amp	-	Morita et al., 2007 [84]
pCAG-JEprME	-	Amp	-	Suzuki et al., 2014 [83]
pCAG-JEprME-HiBiT	pCAG-MCS2	Amp	KpnI/XhoI	-
pCAG-JEprME-D67N (E)-HiBiT	pCAG-MCS2	Amp	KpnI/XhoI	-
pCAG-JEprME-N154A (E)-HiBiT	pCAG-MCS2	Amp	KpnI/XhoI	-
pCAG-JEprME-D67N/N154A (E)-HiBiT	pCAG-MCS2	Amp	KpnI/XhoI	-
pCAG-JEprME-N15A/T17A (prM)-HiBiT	pCAG-MCS2	Amp	KpnI/XhoI	-
pCAG-JEprME-N15A/T17A (prM) + N154A (E)-HiBiT	pCAG-MCS2	Amp	KpnI/XhoI	-
pCAG-JEprME-S275-GS-GFP11-GS-S276	pCAG-MCS2	Amp	KpnI/XhoI	-
pCAG-WNV (NY99) prME-HiBiT	pCAG-MCS2	Amp	KpnI/XhoI	GenBank: DQ211652.1
pCAG-WNV (kunjin) prME-HiBiT	pCAG-MCS2	Amp	KpnI/XhoI	GenBank: KX394384.1
pCAG-YFV (17D) prME-HiBiT	pCAG-MCS2	Amp	KpnI/XhoI	GenBank: JX949181
pCAG-YFV (ASIBI) prME-HiBiT	pCAG-MCS2	Amp	KpnI/XhoI	GenBank: AY640589
pCMV (Δ4.5p)-JErep-nluc	-	Amp	-	Tabata et al., 2021 [85]
pCMV (Δ4.5p)-JErep-nluc-fs	-	Amp	-	Tabata et al., 2021 [85]
pQCGFP1-10er	pQCxIN	Amp	Gibson Assembly	This study.
Str-KDEL_IL-2ss-HiBiT-SBP-GFP1-10 (V206K)	Str-KDEL_TNF-SBP-EGFP	Amp	AscI/PacI	-
pCAG-JEV-5’UTR-C	pCAG-MCS2	Amp	KpnI/XhoI	-
pUC19-JEV-NS1-NS2B	pUC19	Amp	Gibson Assembly	-
pUC19-JEV-NS3-NS4B	pUC19	Amp	Gibson Assembly	-
pCAG-JEV-NS5-3’UTR	pCAG-MCS2	Amp	Gibson Assembly	-
pUC-CPER cassette	pUC19	Amp	Gibson Assembly	-

The Str-KDEL_IL-2ss-HiBiT-SBP-GFP1-10 (V206K) construct was created from the ER-hook-containing Str-KDEL_TNF-SBP-EGFP plasmid [67] (plasmid # 65278; Addgene), in which the TNF-SBP-EGFP sequence was replaced with a tandem sequence of the interleukin-2 signal peptide-HiBiT tag-SBP-GFP1-10 (V206K) at the AscI/PacI site.

### Preparation of recombinant viruses

Recombinant JEVs were generated using the circular polymerase extension reaction (CPER) method with modifications following the procedure outlined in a previous study [63]. The entire genome of JEV strain Nakayama [83], along with its untranslated region (UTR) linkers, was cloned into pUC19 or pCAG-MCS2 vectors into five fragments of cDNA. Mutations of interest were introduced into these plasmids using overlap extension PCR. Six PCR fragments were prepared and subjected to CPER for each assembly. Assembled CPER products were purified and directly transfected into 293T cells. Passage 0 cell culture supernatants were harvested after a certain period.

### Focus-forming assay

Vero cell monolayers were seeded onto 96-well plates and subsequently infected with serial 10-fold dilutions of virus-containing samples in the presence of culture medium. The infected cells were then overlaid with a 1% (w/v) methylcellulose-supplemented medium. After 36 h, the cells were fixed with 4% paraformaldehyde in PBS and permeabilized with 0.1% Triton X-100 and 10% FCS in PBS. Next, the cells were incubated with rabbit polyclonal anti-JEV NS3 antibody, generated against the recombinant JEV C-terminal region of NS3 (aa 1652–2093), for 1 h at 25°C. The cells were then incubated with Alexa Fluor 488-conjugated anti-rabbit antibodies (Jackson ImmunoResearch, West Grove, PA, USA) in PBS for 1 h at 25°C. Finally, virus-positive foci were visualized by fluorescence microscopy and quantified to determine viral infectious titers, expressed as focus-forming units per milliliter.

### Deglycosylation assay

The deglycosylation assay was conducted as described in a previous study with minor modifications [86]. The infected cells were pelleted by centrifugation at 500 × g for 5 min, detached, and resuspended in solubilization buffer containing 20 mM Tris-HCl (pH 7.5), 50 mM NaCl, 10 mM magnesium acetate, 1% Triton X-100, and an EDTA-free protease inhibitor cocktail (Roche Diagnostics, Indianapolis, IN, USA). Supernatants of the infected cells were collected, clarified by centrifugation at 500 × g for 10 min, concentrated at 100,000 × g for 70 min, and resuspended in a solubilization buffer. After the samples had been solubilized by rotating the tube at 4°C for 1.5 h, they were centrifuged at 20,000 × g and 4°C for 15 min. The clarified supernatants were then treated with 12,500 units/mL of PNGase F (New England BioLabs, Ipswich, MA, USA), 12,500 units/mL of Endo H (New England BioLabs), or PBS at 37°C for 4 h in accordance with the manufacturer’s instructions. Finally, the samples were mixed with the 5x sodium dodecyl sulfate (SDS)-PAGE sample buffer, heated at 95°C for 10 min and analyzed by SDS-PAGE, followed by immunoblotting.

### HiBiT detection

Culture supernatants of 293T cells after 48 hours post-transfection were collected and clarified at 500 × g for 10 min. The cell pellet was resuspended in PBS. A Nano-Glo HiBiT Lytic Detection System (Promega, Madison, WI, USA) and a Varioskan LUX Multimode microplate reader (Thermo Fisher Scientific) were used to detect the HiBiT tag in the cells and 500 g of the supernatant, following the manufacturer’s protocol.

### Immunoblotting

The cells were lysed directly using a 2x SDS-PAGE sample buffer and boiled at 95°C for 10 min. Culture supernatants were clarified by centrifugation at 500 × g for 10 min and ultracentrifuged at 100,000 × g at 4°C for 70 min to obtain pellets (100K pellet), which were resuspended in 2x SDS-PAGE sample buffer and boiled. Proteins were separated on 8% or 10% WIDE RANGE polyacrylamide gel (Nacalai Tesque, Kyoto, Japan) and electrically transferred onto polyvinylidene difluoride (PVDF) membranes (Immobilon-P; Merck KGaA, Darmstadt, Germany). Subsequently, the membranes were blocked with 3% skim milk in 0.05% Tween-20/TBS and then incubated with primary antibodies overnight at 4°C. Following this, they were incubated with secondary antibodies for 30 min at 25°C. All relevant antibodies and their dilution rates are listed in Table 2. Proteins were detected using the EzWestLumi plus system (ATTO Technology, Amherst, NY, USA) and a chemiluminescence detector (iBRIGHT CL1000; Thermo Fisher Scientific). To detect the HiBiT-fused envelope protein, the protein-blotted Immobilon-PVDF membrane was probed with bacterially expressed recombinant LgBiT and NanoLuc substrate of the Nano-Glo Luciferase Assay System (Promega), and the luminescence signals were subsequently detected.

**Table 2:** Antibody list.

target	Host	Clone #	Dilution Factor	Conjugation	Experiment	Source	Identifier
tubulin	mouse	AA-4.3	1:1000	none	IB	DSHB	
JEV E	rabbit		1:1000	none	IB	GeneTex	GTX125867
JEV E	mouse	6B4A-10	1:1000	none	IF	Merck Millipore	MAB8743
JEV prM	rabbit		1:1000	none	IB	GeneTex	GTX131833
GM130	mouse	35/GM130	1:1000	none	IF	BD Biosciences	610822
anti-mouse IgG	goat		1:1000	Alexa Fluor 594	IF	Jackson ImmunoResearch	115-585-146
anti-rabbit IgG	goat		1:5000	peroxidase	IB	Jackson ImmunoResearch	111-035-003
anti-mouse IgG	goat		1:5000	peroxidase	IB	Jackson ImmunoResearch	115-035-003

### Indirect immunostaining and fluorescence microscopy

The cells were cultured on coverslips and subsequently fixed in 4% paraformaldehyde in PBS for 10 min at 25°C. Following fixation, cells were permeabilized with 0.1% Triton X-100 in PBS supplemented with 10% FCS for 10 min at 25°C. The cells were incubated with primary antibodies diluted in PBS for 60 min at room temperature, followed by incubation with secondary antibodies diluted in PBS for 60 min at room temperature. A complete list of all antibodies is provided in Table 2. Finally, the coverslips were mounted on slides with Fluoromount-G (SouthernBiotech, Birmingham, AL, USA) and examined using a FluoView FV3000 confocal laser scanning microscope (CLSM) (Olympus, Tokyo, Japan) equipped with a UPLSAPO 60×/1.35 numerical-aperture (NA) oil immersion objective (Olympus). Colocalization quantification of fluorescence signal was performed using the Fiji EzColocalization plugin.

### Retention using selective hooks (RUSH) transport assay

HeLa cells were cultivated in a 35 mm glass bottom dish and incubated in phenol red-free DMEM (Nacalai Tesque) supplemented with 10% FBS, 1 mM sodium pyruvate, and 2 mM L-glutamine for 24 h prior to live imaging or fixation. The release of the RUSH reporters was stimulated by the addition of biotin at a final concentration of 400 μM, which enhanced the synchronization of exports. For time-lapse imaging, widefield microscopy was used to acquire images of the cells using an Olympus IX83 inverted fluorescence microscope equipped with a UPLSAPO 60 × /1.35 numerical-aperture oil immersion objective, and the images were captured with a DP80 camera (Olympus).

### Flow cytometry

To evaluate the GFP fluorescence, HeLa cells were suspended in PBS and analyzed using a CytoFLEX S flow cytometer (Beckman Coulter, Brea, CA, USA).

### Sucrose density gradient analysis

The cell culture supernatant, which was clarified at 500 × g for 10 min, or the cell lysate, which was prepared by a freeze-thaw cycle clarified by centrifugation at 20,000 × g for 10 min, was loaded onto a linear sucrose density gradient of 10–40% (w/v) in PBS. Subsequently, the gradient was centrifuged at 220,000 × g for 3 h at 4°C, and the fractions were collected from the top. The HiBiT tag in each fraction was detected by luminescence and HiBiT blotting as previously described.

### Cycloheximide chase analysis

To assess the degradation of JEV E protein, cells were treated with 50 μg/ml of CHX and then lysed using a 2 × SDS sample buffer at various time points. To determine whether the degradation was mediated by the proteasome, we added 10 μM of MG132, a proteasome inhibitor, to test for inhibition of degradation. SDS-PAGE and immunoblotting were performed using antibodies specific to each protein. Band intensities were measured using ImageJ/Fiji software.

### Statistical analysis

The data are presented as averages ± standard deviations (SDs). Statistical analyses were conducted using the GraphPad Prism v7.0 software (GraphPad Software Inc., La Jolla, CA, USA). Statistical significance was assessed using one-way analysis of variance (ANOVA), followed by either Dunnett’s test for comparison with the control, Tukey’s multiple comparison test for comparison with all groups, or the unpaired t-test to compare the means.
